# Hematological Phenotype of COVID-19-Induced Coagulopathy: Far from Typical Sepsis-Induced Coagulopathy

**DOI:** 10.3390/jcm9092875

**Published:** 2020-09-05

**Authors:** Yutaka Umemura, Kazuma Yamakawa, Takeyuki Kiguchi, Takeshi Nishida, Masahiro Kawada, Satoshi Fujimi

**Affiliations:** 1 Division of Trauma and Surgical Critical Care, Osaka General Medical Center, 3-1-56 Bandai-Higashi, Sumiyoshi, Osaka 558-8558, Japan; take_yuki888@yahoo.co.jp (T.K.); tnishida.716@gmail.com (T.N.); tgv0928@gmail.com (M.K.); sfujimi40@nifty.com (S.F.); 2 Department of Traumatology and Acute Critical Medicine, Osaka University Graduate School of Medicine, Osaka 565-0871, Japan; 3 Department of Emergency Medicine, Osaka Medical College, Osaka 558-8558, Japan; kyamakawa-osk@umin.ac.jp

**Keywords:** COVID-19, sepsis, acute respiratory distress syndrome, blood coagulation disorders, biomarkers

## Abstract

Background: Blood coagulation disorders commonly occur with severe coronavirus disease 2019 (COVID-19). However, there is only limited evidence on differentiating the pattern of the hemostatic parameters from those of typical sepsis-induced coagulopathy (SIC). Methods: To elucidate the specific pattern of coagulopathy induced by COVID-19 pneumonia, this retrospective, observational study targeted consecutive adult patients with COVID-19-induced acute respiratory distress syndrome (ARDS) and compared hemostatic biomarkers with non-COVID-19-induced septic ARDS. Multilevel mixed-effects regression analysis was performed and Kaplan–Meier failure curves were constructed. Results: We enrolled 24 patients with COVID-19-induced ARDS and 200 patients with non-COVID-19-induced ARDS. Platelet count, antithrombin activity, and prothrombin time in the COVID-19 group were almost within normal range and time series alterations of these markers were significantly milder than the non-COVID-19 group (*p* = 0.052, 0.037, and 0.005, respectively). However, fibrin/fibrinogen degradation product and D-dimer were significantly higher in the COVID-19 group (*p* = 0.001, 0.002, respectively). COVID-19 patients had moderately high levels of thrombin–antithrombin complex and plasmin-alpha2-plasmin inhibitor complex but normal plasminogen activator inhibitor-1 level. Conclusions: The hematological phenotype of COVID-19-induced coagulopathy is quite different from that in typical SIC characterized by systemic hypercoagulation and suppressed fibrinolysis. Instead, local thrombus formation might be promoted in severe COVID-19.

## 1. Introduction

The novel corona virus infection (coronavirus disease 2019: COVID-19), originating in Wuhan, China, has rapidly spread worldwide [1]. As of July 15, 2020, more than 13.8 million cases and approximately 590,000 deaths were reported from all over the world.

Along with acute respiratory distress syndrome (ARDS), coagulation disorders are reported to be induced by severe COVID-19 pneumonia and to be associated with increased risk of death [2,3,4,5]. Besides, a previous study reported that severe acute respiratory syndrome coronavirus 2 (SARS-CoV-2) could directly infect the vascular endothelial cells and facilitate the induction of vascular endothelial dysfunction. [6] Endothelial dysfunction and platelet activation induced by acute inflammation are ubiquitous in COVID-19-associated coagulopathy and might play key roles in the progression of organ ischemia and subsequent death [7].

In this context, several anticoagulant or thrombolytic agents are expected to be beneficial in the treatment of COVID-19 infections [8,9]. However, the best management for coagulopathy in COVID-19 remains a matter of dispute, partly due to the limited evidence on the clinical features of COVID-19-induced coagulopathy. Phenotypes of coagulopathy and the expected efficacy of therapies vary widely according to the underlying diseases [10,11]. Therefore, precise knowledge of the alteration of hemostatic parameters and the incidence of coagulopathy in severe COVID-19 patients is required to consider appropriate management, especially in patients admitted to the intensive care unit (ICU) who are at high risk for thrombotic complications. So far, only limited information has been available on the alteration of hemostatic parameters induced by severe COVID-19 infections, and it is not fully understood whether “the COVID-19-induced coagulopathy” is a specific phenotype of coagulation disorder or merely one type of sepsis-induced coagulopathy (SIC).

Herein, we aimed to elucidate the specific pattern of coagulopathy in severe COVID-19 pneumonia by comparing it with the patterns in patients with non-COVID-19-induced severe pneumonia.

## 2. Experimental Section

### 2.1. Study Design, Setting, and Participants

This was a single-center, retrospective, observational study conducted at a tertiary care hospital in Japan. All adult patients who were admitted to the ICU with COVID-19-induced ARDS and required mechanical ventilation during March 1 through April 30 in 2020 were consecutively enrolled in this study. As a control group, we included consecutive adult patients who were admitted to the ICU with the diagnosis of septic ARDS induced by non-COVID-19 community-acquired bacterial pneumonia and required mechanical ventilation from January 2013 to February 2020.

The exclusion criteria included the use of warfarin/acetylsalicylic acid/thrombolytic therapy before study entry; the limitation of sustained life care or post-cardiopulmonary arrest resuscitation status; history of fulminant hepatitis, decompensated liver cirrhosis, or other serious liver disorder; history of hematologic malignant disease; and other conditions increasing the risk of thrombosis at study entry.

This study followed the principles of the Declaration of Helsinki. The protocol was approved by the Institutional Review Board for Clinical Research of Osaka General Medical Center (IRB No. S202004004).

### 2.2. Diagnosis of COVID-19 and ARDS

The diagnosis of COVID-19 was performed according to World Health Organization interim guidance [12] and confirmed by RNA detection of SARS-CoV-2 in a clinical laboratory of the Osaka Institute of Public Health. In this study, sepsis was diagnosed based on Sepsis-3 criteria proposed in 2016 [13]. ARDS was diagnosed according to the Berlin definition [14] as fulfilling the following criteria: (1) onset within 1 week after predisposing diseases, (2) bilateral infiltration on chest roentgenogram, (3) PaO_2_/FIO_2_ (P/F ratio) ≤ 300 with PEEP ≥ 5 cm H_2_O, and (4) no clinical signs of cardiac failure or fluid overload. We defined the first day of mechanical ventilation as “day 1” both in the COVID and non-COVID pneumonia groups.

### 2.3. Data Collection

Patients were followed up until hospital discharge or death. Patient information collected included demographic characteristics, pre-existing comorbidities, laboratory tests, severity scores, and therapeutic interventions.

Laboratory tests included several hemostatic biomarkers, such as platelet count, prothrombin time (PT), fibrinogen level, fibrin/fibrinogen degradation products (FDPs), D-dimer level, antithrombin activity, thrombin–antithrombin (TAT) complex, plasmin-a2-plasmin inhibitor complex (PIC), and plasminogen activator inhibitor (PAI)-1. TAT, PIC, and PAI-1 were evaluated only on day 1 in the COVID-19 group; however, the other biomarkers were measured in both groups at each time point from day 1 to day 7.

Severity of illness was evaluated according to the Sequential Organ Failure Assessment (SOFA) score and the Acute Physiology and Chronic Health Evaluation (APACHE) II score. The APACHE II score was evaluated on day 1, and the SOFA score was evaluated at each time point from day 1 to day 7.

The incidence of disseminated intravascular coagulation (DIC) was evaluated at each time point from day 1 to day 7 based on the International Society on Thrombosis and Hemostasis (ISTH) overt DIC and the Japanese Association for Acute Medicine (JAAM) DIC criteria. We also evaluated the incidence of SIC at each time point [15]. The ISTH overt DIC scoring system was adopted as proposed by the Scientific Subcommittee on DIC of the ISTH for platelet counts, PT, FDP, and fibrinogen level [16]. To calculate the ISTH overt DIC score, FDP values were chosen as the fibrin-related marker and scored according to the cut-off levels and ranges previously published by Gando et al. [17]. The JAAM DIC scoring system consists of the SIRS (systemic inflammatory response syndrome) score and global coagulation tests including platelet counts, PT, and FDP/D-dimer levels. [18] We used prophylactic low-dose unfractionated heparin for both the COVID-19 and control groups when patients had a high risk of venous thromboembolism (VTE), such as severe obesity, cancer, orthopedic surgery, and prior history of VTE.

### 2.4. Statistical Analysis

The aim of this study was to reveal the specific pattern of coagulopathy induced by severe COVID-19 pneumonia by comparing the hemostatic parameters chronologically with those in patients with ARDS induced by non-COVID-19 pneumonia. Therefore, to assess the time series variation in the hemostatic parameters, we fitted multilevel mixed-effects regression models with fixed effects assigned to patient categorization (COVID-19 or non-COVID-19), time points (from day 1 to day 7), and two-way interaction term for these independent variables, and random effects assigned to individual identification numbers. We also performed multilevel mixed-effects regression analysis to evaluate the time series differences during the first seven days in other organ dysfunction parameters, including P/F ratio, serum creatinine level, serum bilirubin level, Glasgow Coma Scale, and SOFA subscore for the cardiovascular component between the COVID-19 and non-COVID-19 groups.

Kaplan–Meier failure curves were constructed to evaluate the cumulative incidence of JAAM DIC, ISTH overt DIC, and SIC over time (from day 1 to day 7). Log rank tests were conducted to compare the Kaplan–Meier curves between two groups.

Descriptive statistics were calculated as medians (interquartile range) or proportions (numbers), as appropriate. Univariate differences between groups were assessed using the Mann–Whitney U test or chi-square test, as appropriate. Missing values were not imputed in any of the regression models.

All statistical inferences were performed with a two-sided *p* at the 5% significance level. Because of the underpowered nature of the interaction analysis, we used a two-sided significance level of 20% with statistical inferences for the interaction analyses [19]. All statistical analyses were conducted using STATA Data Analysis and Statistical Software version 15.0 (StataCorp, College Station, TX).

## 3. Results

### 3.1. Study Population

**Figure 1:**
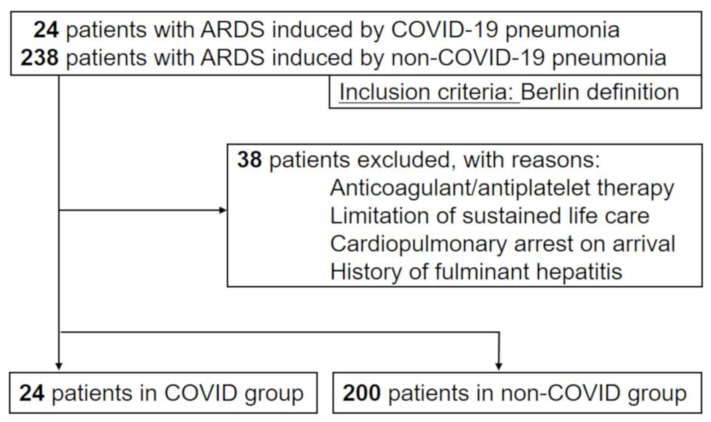
Patient flow diagram. ARDS, acute respiratory distress syndrome; COVID-19, coronavirus disease 2019.

From March to May 2020, 24 consecutive patients were admitted to the ICU with the diagnosis of ARDS induced by COVID-19 and underwent mechanical ventilation. Also, 238 patients with ARDS induced by non-COVID-19 bacterial pneumonia who fulfilled our inclusion criteria from January 2013 to February 2020 were included. After excluding 38 patients who met our exclusion criteria, we analyzed 24 patients as the COVID-19 group and 200 patients as the non-COVID-19 group (Figure 1).

Baseline characteristics, laboratory tests, and illness severity scores on day 1 in the two groups are shown in Table 1. Age, sex, and pre-existing comorbidities were similar between the groups. Although the platelet counts on day 1 were similar between the two groups, there were statistically significant differences in other hemostatic biomarkers. PT (%), fibrinogen level, and antithrombin activity in the COVID-19 group were almost within the normal range and were significantly higher compared with those in the non-COVID-19 group.

There were no significant differences between groups in mental status and respiratory function, as indicated by the Glasgow Coma Scale and P/F ratio, respectively. DIC scores and SIC score were significantly lower in the COVID-19 group compared with those in the non-COVID-19 group.

### 3.2. Hemostatic Parameters in COVID-19 Pneumonia

**Figure 2:**
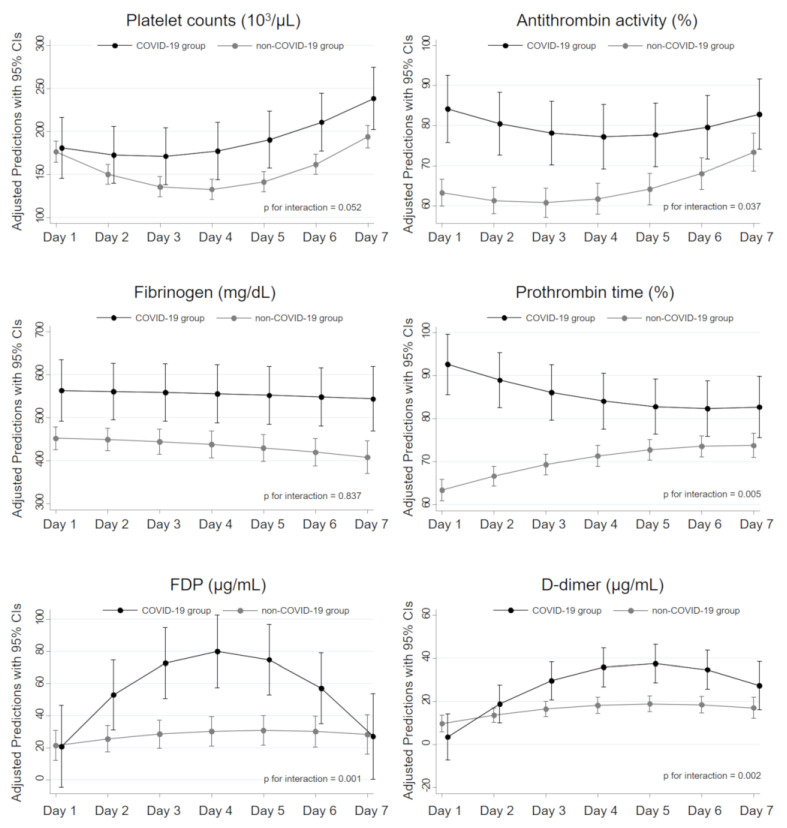
Time series differences in hemostatic parameters between the COVID-19 and non-COVID-19 groups. The regression line with 95% confidence intervals (CIs) in each group was estimated by a multilevel mixed-effects regression model with a two-way interaction term between the group and time series. The solid black lines represent patients in the COVID-19 group, and the solid gray lines those in the non-COVID-19 group. COVID-19, coronavirus disease 2019; FDP, fibrin/fibrinogen degradation products.

A multilevel mixed-effects regression model suggested that the changes in platelet counts over time during the first seven days were significantly different between the two groups (*p* for interaction = 0.052), and the COVID-19 group had constantly higher platelet counts after day 2 (Figure 2). Similarly, we detected significant time series differences in PT, FDP, D-dimer, and antithrombin activity between the two groups (*p* for interaction = 0.005, 0.001, 0.002, and 0.037, respectively). The COVID-19 group had higher levels (almost within normal range) of PT and antithrombin activity at all day points but higher levels of FDP and D-dimer after day 2.

**Figure 3:**
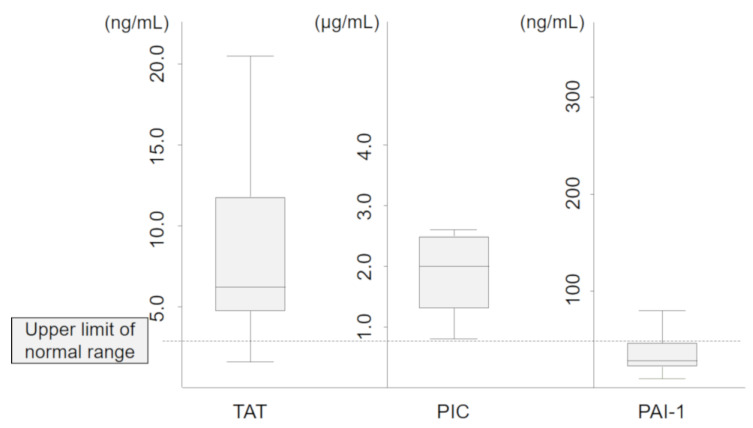
Hemostatic molecular biomarkers in the COVID-19 group. The dotted line represents the upper limit of normal range of each marker. TAT, thrombin antithrombin; PIC, plasmin-a2-plasmin inhibitor complex; PAI, plasminogen activator inhibitor.

We also show the levels of several hemostatic molecular biomarkers, such as TAT, PIC, and PAI-1, in the COVID-19 group in Figure 3. The median levels of TAT and PIC were 6.8 ng/mL and 2.0 μg/mL, respectively, and tended to be higher compared with the upper limits of normal range for these two biomarkers (3.0 ng/mL and 0.8 μg/mL, respectively). However, the median level of PAI-1 was 28.0 ng/mL and was within the normal range (<50.0 ng/mL).

### 3.3. Differences in Clinical Manifestations over Time

**Figure 4:**
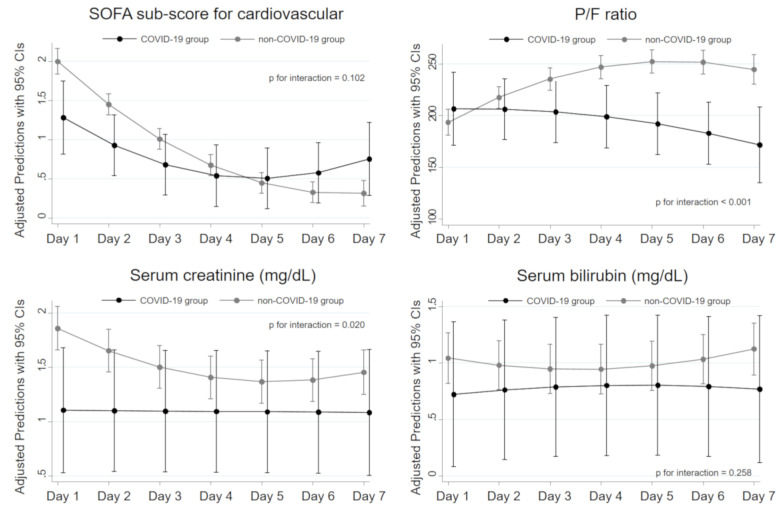
Time series differences in organ dysfunction parameters between the COVID-19 and non-COVID-19 groups. The regression line with 95% confidence intervals (CIs) in each group was estimated by a multilevel mixed-effects regression model with a two-way interaction term between the group and time series. The solid black lines represent patients in the COVID-19 group, and the solid gray lines those in the non-COVID-19 group. SOFA, Sequential Organ Failure Assessment; P/F, PaO_2_/FIO_2_; COVID-19, coronavirus disease 2019.

We show the time series differences in several parameters related to organ dysfunction other than coagulation in Figure 4. Although the P/F ratio in the non-COVID-19 patients improved with time, that in the COVID-19 group worsened with time, and there were significant time series differences between the two groups (*p* for interaction < 0.001). In contrast, serum levels of creatinine tended to be lower at all time points in the COVID-19 group. Only slight differences were detected in serum bilirubin level and the SOFA subscore for the cardiovascular component between the COVID-19 and non-COVID-19 groups.

### 3.4. Incidence of DIC

**Figure 5:**
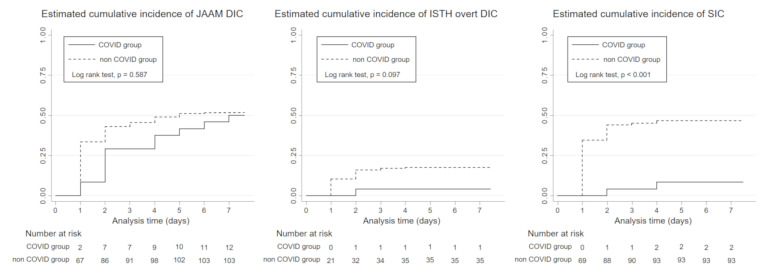
Cumulative incidence of JAAM DIC, ISTH overt DIC, and SIC in the COVID and non-COVID groups during the first seven days from the initiation of mechanical ventilation. The solid lines represent patients in the COVID-19 group, and the dotted lines those in the non-COVID-19 group. COVID-19, coronavirus disease 2019; JAAM, Japanese Association for Acute Medicine; DIC, disseminated intravascular coagulation; ISTH, International Society on Thrombosis and Hemostasis; SIC, sepsis-induced coagulopathy.

The cumulative incidence curve constructed with the Kaplan–Meier method showed that there were no statistically significant differences in the incidence of JAAM DIC and ISTH-overt DIC during the first seven days between the groups (log rank test, *p* = 0.587 and 0.101, respectively). However, the incidence of SIC in the COVID-19 group during the first seven days was only 8.5% (2 of 24 patients) and was significantly lower compared with that in the non-COVID-19 group (log rank test, *p* = 0.003, Figure 5). One of the two SIC patients survived to hospital discharge, however, another patient developed multiple organ dysfunction syndrome and died despite full intensive care support.

## 4. Discussion

To date, several studies have reported on COVID-19-related coagulopathy [2,5,20,21]; however, in terms of the phenotypes or incidence of DIC, there remains little evidence about whether it can be differentiated from overall SIC. In the present study, we compared the pattern and incidence of coagulopathy over time between severe COVID-19 pneumonia and non-COVID-19-induced severe pneumonia using multilevel time series analyses and the Kaplan–Meier method.

### 4.1. Specific Phenotype of Coagulopathy in COVID-19

A recent study reported that the baseline levels of platelet count, PT, antithrombin activity, and fibrinogen were significantly higher in COVID-19 patients compared to patients with non-COVID-19 ARDS [22]. A similar pattern of hemostatic markers was observed in the present analysis at day 1: PT, antithrombin activity, and fibrinogen in the COVID-19 group were almost within normal range and significantly higher compared with the values in the non-COVID-19 group. Furthermore, we showed that the chronological alterations of platelet count, antithrombin activity, and PT in the COVID-19 group were significantly milder, whereas elevations of FDP and D-dimer were significantly higher in the COVID-19 group.

Decreased platelet counts and prolonged prothrombin time are the most common hematologic signs induced by systemic hypercoagulation and therefore are involved in all widely used DIC criteria [15,16,18]. Antithrombin activity is also known to be markedly decreased in sepsis-induced coagulation, due to consumption as a result of ongoing thrombin generation [23]. However, elevations of FDP and D-dimer are generally within a mild range in the early phase of SIC, mainly due to the impaired fibrinolysis driven by an increase in PAI-1 [24,25]. High levels of FDP and D-dimer with normal or mild alterations of other coagulation markers are typical in local thrombus formation, such as pulmonary embolism and deep venous thrombosis [26,27]. Indeed, several studies have reported high incidences of pulmonary embolism in COVID-19 patients with or without underlying deep venous thrombosis [28,29]. According to these insights, our results suggested that systemic hypercoagulation was hardly induced by COVID-19 infection, but the risk of local thrombus formation increased in the acute phase of severe COVID-19 pneumonia.

We also evaluated several hemostatic biomarkers and found that COVID-19 patients had moderately high levels of TAT and PIC but a normal level of PAI-1. Vascular endothelial cell dysfunction is an essential feature in the pathogenesis of SIC. Because the secretion of PAI-1 is mainly regulated by endothelial cells, sepsis-related endothelial cell dysfunction causes a marked increase in the PAI-1 level leading to disrupted fibrinolysis, and this key event represents the typical feature of the thrombotic type of DIC [30]. Therefore, our results regarding the levels of PAI-1 suggested that vascular endothelial cell dysfunction and the thrombotic type of DIC were hardly induced by severe COVID-19 pneumonia.

Then, what phenotype of coagulopathy is induced by COVID-19 pneumonia? Severe coagulopathy can be classified into three common phenotypes according to the underlying disease: (1) enhanced fibrinolytic phenotype typically induced by acute promyelocytic leukemia, (2) balanced fibrinolytic phenotype typically induced by cancer, and (3) suppressed fibrinolytic phenotype typically induced by sepsis. In the present study, patients with COVID-19 pneumonia had moderate elevations of TAT and PIC and a normal level of PAI-1, which are characteristic of the balanced fibrinolytic phenotype [10]. One possible explanation for this specific phenotype of coagulopathy is that a pulmonary-restricted intravascular coagulopathy initially occurs due to the extensive alveolar and interstitial inflammation that occurs in patients with COVID-19 pneumonia, and this causes the expression of active tissue factor leading to balanced fibrinolytic coagulopathy [31].

### 4.2. Incidence of DIC in COVID-19

As mentioned above, the marked elevation of fibrin degradation products was a main characteristic of COVID-19-induced coagulopathy. Therefore, the incidence of JAAM DIC and ISTH overt DIC involving the component of fibrin degradation products was equal between the two groups during the first seven days, whereas the incidence of SIC, which does not involve the component of fibrin degradation products, was much lower in the COVID-19 group. These findings suggested that the typical coagulopathy in COVID-19 was distinct from SIC, even though it could meet several of the DIC criteria.

### 4.3. Limitations

We acknowledge several limitations of our study. First, the single-center design and short study duration resulted in a relatively small sample size, which may have influenced the precision of our findings. Second, we enrolled patients with different pathophysiology (bacterial ARDS) as the control group for the study purpose of evaluating the specific phenotype of severe COVID-19-induced coagulopathy by comparing it to other types of sepsis. As a result, there were no differences in baseline characteristics and pre-existing comorbidities, but the pattern of organ dysfunction between the groups reflected the unique clinical manifestation of severe COVID-19 pneumonia. However, we are not confident that the effect of potential ascertainment bias can be completely excluded despite robust adjustment with regression models. Third, the long-term design of the control group might be another source of bias. Although the type and severity of patients and the key concepts for the management of ARDS were not greatly changed, physician staffing and several supportive therapies, such as antimicrobials and nutrition, might change according to the time course, which possibly influenced the study findings. Finally, the control group included only bacterial sepsis and did not include non-COVID-19 viral infections. Further investigation is thus required to compare the coagulation disorders between COVID-19-induced ARDS and non-COVID-19-viral-infection-induced ARDS.

## 5. Conclusions

In the present study, the pattern of coagulopathy in severe COVID-19 pneumonia was quite different from that in other severe pneumonias. Systemic hypercoagulation, suppressed fibrinolysis, and vascular endothelial cell dysfunction, typically observed in sepsis-induced DIC, might be hardly induced; instead, local thrombus formation was possibly triggered by pulmonary-restricted intravascular coagulopathy in COVID-19 pneumonia. Further investigations are required to confirm our findings and establish appropriate management for severe COVID-19 pneumonia.

## Figures and Tables

**Table 1:** Baseline characteristics, laboratory tests, and severity scores in study population.

	COVID-19 Group	Non-COVID-19 Group	*p* Value
	*n* = 24	*n* = 200	
Sex (male)	15 (63%)	138 (69%)	0.518
Age (year)	71 (64–76)	72 (66–79)	0.336
Pre-existing conditions			
Hypertension	10 (42%)	51 (26%)	0.093
Diabetes mellitus	10 (42%)	59 (30%)	0.223
Chronic heart failure	2 (8%)	27 (14%)	0.382
Chronic kidney disease	2 (8%)	24 (12%)	0.596
Chronic liver failure	2 (8%)	17 (9%)	0.978
Chronic respiratory failure	3 (13%)	30 (15%)	0.744
Cerebrovascular disease	4 (17%)	29 (15%)	0.777
Cancer	2(8%)	12 (6%)	0.317
Pathogens			
Gram-positive	-	33 (16.5%)	
Gram-negative	-	71 (35.5%)	
Polymicrobial infection	-	63 (31.5%)	
Culture negative	-	33 (16.5%)	
Laboratory tests			
Platelet count (10^3^/µL)	177 (125–249)	173 (117–246)	0.905
Prothrombin time (%)	90.6 (83.4–98.3)	65.7 (50.1–79.1)	<0.001
Fibrinogen (mg/dL)	577.5 (500.5–672.5)	429 (304–558)	<0.001
Antithrombin activity (%)	85.1 (75–95.5)	63 (51.6–77.7)	<0.001
FDP (µg/mL)	5.1 (3.8–7.8)	10.6 (5.6–21.7)	0.001
D-dimer (µg/mL)	2 (1.2–5)	4.4 (2.1–11.5)	0.006
Serum creatinine (mg/dL)	0.9 (0.6–1.1)	1.3 (0.8–2.2)	0.001
Serum bilirubin (mg/dL)	0.6 (0.5–0.9)	0.8 (0.5–1.2)	0.139
CRP (mg/dL)	14.2 (9.1–20.3)	10.7 (4.4–19.9)	0.157
White blood cells (10^3^/µL)	6.3 (5.1-8)	7.8 (4.1–11.8)	0.154
Severity of illness			
Glasgow Coma Scale	13 (6–15)	11 (6–14)	0.631
P/F ratio	181 (124–277)	176 (121–256)	0.417
SOFA score	7 (5–8)	8 (6–11)	0.012
APACHE II score	19 (12–25)	22 (16–27)	0.105
JAAM DIC score	0.5 (0–1)	2 (1–4)	<0.001
ISTH DIC score	0 (0–1)	2 (1–4)	<0.001
SIC score	2 (2–2)	3 (2–4)	<0.001

Abbreviations: COVID-19, coronavirus disease 2019; FDP, fibrin/fibrinogen degradation products; CRP, C-reactive protein; P/F, PaO_2_/FIO_2_; SOFA, Sequential Organ Failure Assessment; APACHE, Acute Physiology and Chronic Health Evaluation; JAAM, Japanese Association for Acute Medicine; DIC, disseminated intravascular coagulation; ISTH, International Society on Thrombosis and Hemostasis; SIC, sepsis-induced coagulopathy.
